# Environmental impact and mobility of thallium and other metal(oid)s in soils and tailings near a decommissioned Zn-Pb mine (Raibl, NE Italian Alps)

**DOI:** 10.1007/s10653-025-02400-4

**Published:** 2025-02-25

**Authors:** Nicolò Barago, Elena Pavoni, Federico Floreani, Matteo Crosera, Gianpiero Adami, Davide Lenaz, Stefano Covelli

**Affiliations:** 1 Department of Mathematics, Informatics and Geosciences, University of Trieste, Via Weiss 2, 34128 Trieste, Italy; 2 Department of Chemical and Pharmaceutical Sciences, University of Trieste, Via Giorgieri 1, 34127 Trieste, Italy

**Keywords:** Thallium, Potentially toxic elements, Environmental impact, Contamination, Soil, Mine wastes

## Abstract

The potential impact of decommissioned mining areas on environmental quality is of major concern for local communities, posing a risk to water resources and human health. This study aims to investigate the impact of extraction activities on the surface environment by evaluating the occurrence of metal(oid)s, including potentially toxic elements (PTEs, i.e. As, Cd, Fe, Tl, Zn, Pb) and critical elements (As, Ge), at the Zn-Pb Raibl mining area (northeastern Italy). Elevated concentrations of metal(oid)s are found near mine waste heaps (< 100 mg/kg for Tl, Sb, Cd, Ge; > 1,000 mg/kg for As; > 1% for Pb and > 10% for Zn and Fe), which are made up of flotation tailings and waste rocks scattered around the mining village and stored in the tailings impoundments. Conversely, upstream from the mine, the environment is largely uncontaminated. According to the results, total and leachable metal(oid) concentrations are positively correlated. Tailings (65.1–754 mg/kg of Tl) are identified as the primary source of leachable Tl (11.4–255 mg/kg) and metal(oid)s are generally more mobile in organic-rich soils, suggesting increased metal(oid) mobility with soil ageing due to low soil pH and potential soluble organometallic complexes. Furthermore, the findings suggest that reprocessing of mine tailings could be a potential solution to recover valuable elements together with residue backfilling. Lastly, results from this study highlight how crucial mining site management is to limit PTE dispersion and reducing risks to the environment and public health.

**Supplementary Information:**

The online version contains supplementary material available at 10.1007/s10653-025-02400-4.

## Introduction

The occurrence of metal(oid)s, including potentially toxic elements (PTEs) such as lead (Pb), zinc (Zn), thallium (Tl), arsenic (As), cadmium (Cd) and antimony (Sb) in the environment as the legacy of past mining activities is a well-known global concern (El Rasafi et al., 2017; Žibret et al., 2018). Due to prolonged exposure to PTEs through ingestion, inhalation, skin contact, people who live and work close to mines face notable health risks (Du et al., 2019; Yang et al., 2015). Understanding geochemical processes is crucial to managing both active and decommissioned mining sites, as well as controlling the dispersion of metal(oid)s by lowering the risk to ecosystems and human health which can persist for decades or centuries in the large mining sites (Cidu et al., 2018; Covelli et al., 2007; De Giudici et al., 2019; Gosar et al., 2020; Gošar et al., 2015; Higueras et al., 2006; Hudson-Edwards, 2003; Kasmaeeyazdi et al., 2022; Pavoni et al., 2017; Wang et al., 2017). As remediation of mining sites is challenging, burdensome and very expensive, a change of perspective is needed, considering the occurrence of metal(oid)s not only as an issue, but also as an opportunity. Indeed, large amounts of mine wastes (such as tailings impoundments), which normally represent the major source of contamination for the surrounding environment, can host valuable amounts of metal(oid)s of economic interest. Some are included in the two lists of materials (34 are listed as “critical” and 17 are listed as “strategic”) which are considered crucial for the EU’s green and digital transitions, and for the defence and space industries (Regulation (EU) 2024/1252). Several of those metal(oid)s could also be recovered from mine wastes and tailings which are often abandoned in ore mining areas which are no longer in operation (Ceniceros-Gómez et al., 2018). In this case, considerable benefits are also expected as a result of the remediation of decommissioned mining sites (Asadollahfardi et al., 2021; Venkateswarlu et al., 2016).

Effective environmental management and remediation strategies from a sanitary and environmental perspective depend on understanding the complex patterns of leaching in mining areas. This is crucial to properly assess the potential transfer of metal(oid)s to the surrounding environmental matrices and through the trophic webs (Alloway, 2013; Khelifi et al., 2020; Teršič et al., 2014). The biogeochemical and ecotoxicological behaviour of metal(oid)s is governed not only by their total concentration but also by their speciation as the various species occurring in the environment may be characterised by different mobility and potential bioavailability. Thus, the evaluation of the labile fraction of metal(oid)s via single or sequential partial extraction (PE) (Kersten, 2002; Pueyo et al., 2008) may be useful to determine labile metal(oid) concentrations that are potentially bioavailable and therefore harmful or toxic to the biota (Botsou et al., 2022; Snape et al., 2004) together with the factors that regulate their mobility. Although the reagents or extractants used in PE are “non-selective” and cannot unequivocally determine the speciation of the elements of interest (i.e. the mineral/organic phase which hosts or binds the metal(oid)s), PE allows one to assess some environmentally relevant characteristics such as the relative amounts of labile and non-labile fractions. Several PE procedures are reported in the literature, and the diluted 0.5 M HCl moderate single step extraction is one of the most commonly adopted for soils and sediments (Adami et al., 1999; Bermudez et al., 2010; Botsou et al., 2022; Kubová et al., 2008; Larner et al., 2006; Madrid et al., 2007; Reis et al., 2015; Salazar et al., 2012; Sutherland, 2002; Sutherland & Tack, 2008). This PE assumes that labile trace metal(oid)s are associated with degradable organic matter, more reactive minerals and with surface coatings of mineral particles resulting in greater availability than those in primary minerals, that is in their crystalline lattice structure (Sutherland et al., 2001 and references therein). It is a valuable and cost-effective analytical procedure for determining labile phases in the framework of metal(oid) contamination assessment in part because it produces minimal dissolution of crystalline lattices of silicates (Agemian & Chau, 1976, 1977; Larner et al., 2006; Sutherland, 2002). This procedure is also a valid and fast alternative to the BCR (European Community Bureau of Reference) sequential extraction (Rauret et al., 1999) since strong correlations were found between the diluted 0.5 M HCl moderate single step extraction and the sum of the three labile steps of the BCR sequential extraction, for instance, in urban soils and road dust (Larner et al., 2006; Sutherland, 2002).

The purpose of this study is to assess the impact on the territory related to the dispersion of contaminated material as a result of past extraction activity within a Zn-Pb mining district (Raibl, NE Italy). More specifically, we intended to identify the most critical areas not only in terms of the total amounts of metal(oid)s but also to investigate the mobility of Tl along with As, Cd, Pb and Zn through a diluted HCl extraction procedure in different solid matrices, from uncontaminated to contaminated soils heavily enriched by mining residues. Results were also evaluated and related to other soil characteristics such as organic matter (OM) content and pH, which can strongly affect metal(oid) mobility.

## Study area and geological setting

**Fig. 1:**
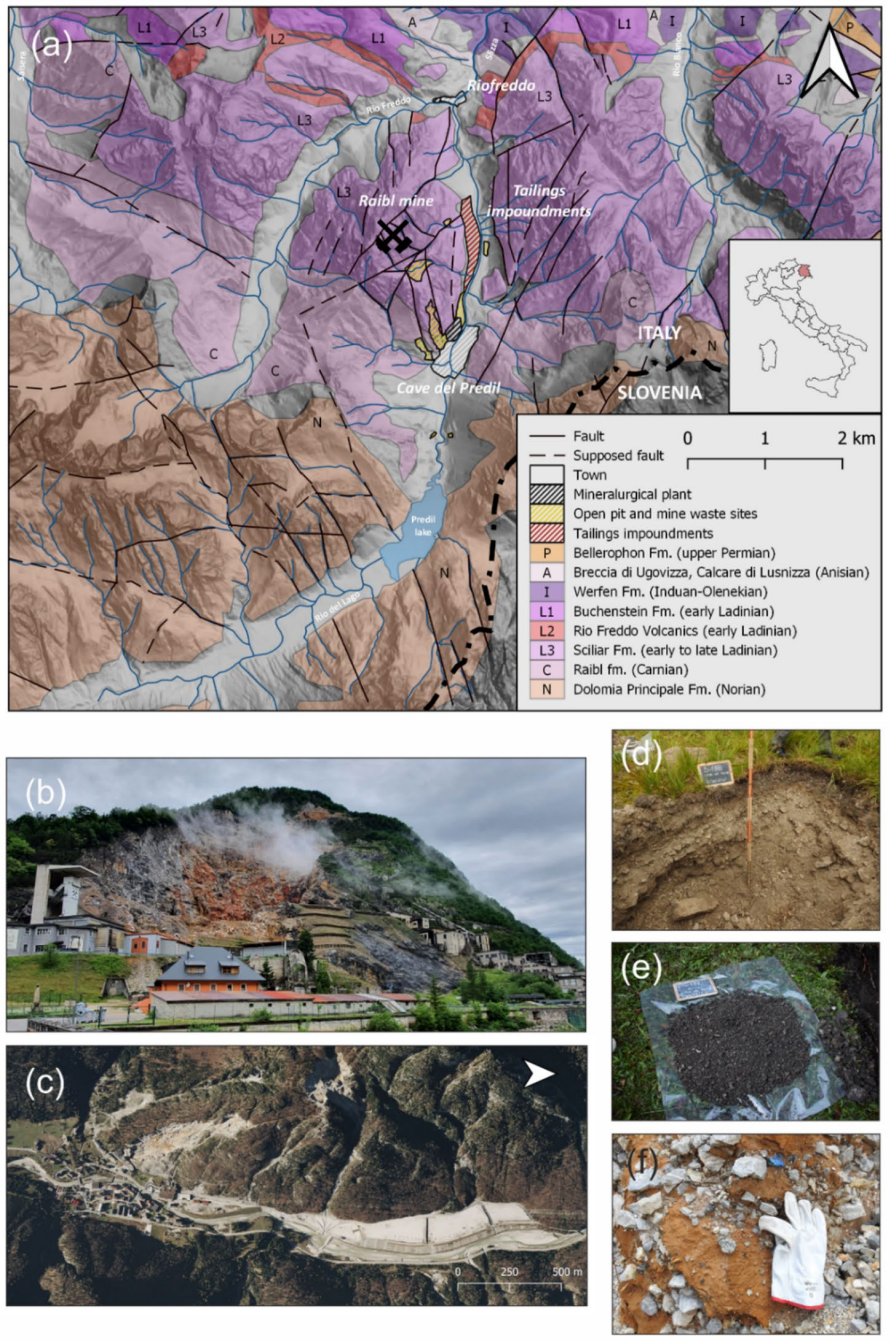
**a** Legend of Triassic lithologies. (B) Bellerophon Fm.: Limestones, dolostones and gypsum; (I) Werfen Fm.: Sandstones, siltstones and marls, calcarenites and limestones; (A) Breccia di Ugovizza and Calcare di Lusnizza Fm.: conglomerates and limestones; (L1) Buchenstein Fm.: limestones, marls, calcarenites, tuffaceous sandstones, siltstones; (L2) Rio Freddo Volcanics: acid to intermediate pyroclastic rocks; (L3) Sciliar Fm.: dolostones and limestones; (C) Raibl fm.: (bituminous) limestones, marls, and mudstones; (N) Dolomia Principale Fm.: Dolostones and limestones (Carulli, 2006). **b** View of the Raibl mine, left: Clara main shaft; right: the metallurgical plant. **c** left: the village of Cave del Predil; right: the tailings impoundments. Examples of **d** soil, **e** waste rock and **f** tailings samples

The Zn-Pb Raibl mine is located near the village of Cave del Predil, at the NE border of the Friuli Venezia Giulia region (NE Italy), in the Julian Alps (Figure 1a). Near the borders with Slovenia, to the east, and Austria, to the north, the mine is located in a mountainous area in and around Mt. Re, where the ore deposit is hosted. The main stream is the Rio del Lago which flows from S to N into the Slizza stream, a tributary of the Gail-Drava-Danube River system flowing into the Black Sea. Both the Rio del Lago, Slizza and Gail Rivers are contaminated by past Zn-Pb mining and metallurgical processing (Barago et al., 2023; Muller et al., 1994).

The Zn-Pb ore deposit classification at the Raibl mine is still disputed, being classified as a Mississippi Valley-Type (MVT) (Leach et al., 2010) or by Austrian and Italian authors as an “Alpine-type” (Brigo et al., 1977; Henjes-Kunst et al., 2017; Schroll, 2005), a sub-type of carbonate-hosted deposits. The paragenesis is simple and includes sphalerite (ZnS), galena (PbS), iron (Fe) sulphides (mainly pyrite and marcasite, FeS_2_), and baryte (BaSO_4_) as primary minerals; included among the secondary minerals which are the product of the oxidation of primary minerals are Hydrous Ferric Oxides (HFOs), smithsonite (ZnCO_3_), hydrozincite (Zn_5_(CO_3_)_2_(OH)_6_) and cerussite (PbCO_3_), with dolomite (CaMg(CO_3_)_2_) and calcite (Ca(CO3)) among the gangue minerals (Di Colbertaldo, 1948). In addition to Zn and Pb, the elements of interest hosted in sulphides are As, Cd, Ge, Sb and Tl.

Raibl, together with the Bleiberg (Austria), Mežica (Slovenia) and Salafossa (Veneto, Italy) mines, constituted one of the most important Zn-Pb mining districts in Europe. On the whole, during the 1960s-70s, they produced more than 75% of the total Pb and Zn of the Alpine area (Brigo et al., 1977). The ore deposit, likely exploited before 1320, the year of the first documented mining activity, was the most important in the Friuli Venezia Giulia region and the last to be decommissioned in 1991 (Zucchini, 1998). Currently, there are no active metal mines in the region. Although Fe was mined in Roman times, Zn, Pb, and germanium (Ge) were mainly mined in the last centuries. The peak production of the Raibl mine occurred in the 1960s, with about 550,000 tons/year of raw ore extracted. The production of Zn concentrate was estimated to be 40,000–45,000 tons/year and Pb concentrate reached about 4,000–5,000 tons/year, obtained via froth flotation in the ore processing plant at Cave del Predil (Figure 1b). Tailings (Figure 1f) were discharged directly into the Rio del Lago until 1976, they were then stored as slurry in the purpose-built tailings impoundments, on the stream bed of the Rio del Lago (Figure 1c), a few hundred metres north of the village of Cave del Predil, until 1991. Most of the tailings, i.e. the fine-grained by-product of the froth flotation, are depleted in sphalerite and galena, but rich in discarded primary and secondary minerals such as HFOs, pyrite, Zn and Pb carbonates, and gangue minerals (Barago, 2023). Secondary minerals were confirmed by SEM analysis on heavy minerals of oxidised material recovered from surface tailings (Barago, 2023, Tab. S1 and Fig. S1), which are similar to the samples analysed in this study. Volume estimations indicate an amount of about 4 million tons of tailings and waste rocks enriched with various elements of environmental concern (i.e. As, Cd, Pb, Sb, Tl and Zn) and/or potential economic interest (Barago et al., 2023; Meriggi et al., 2008) (including Zn, Pb base metals and critical elements for the European Union such as Ge and As) (European Commission, 2020; Barago et al., 2021) accumulated in tailings impoundments. However, since elevated concentrations of Tl, Zn, Pb and Cd were found in groundwater, stream water and sediments (Barago, 2023; Barago et al., 2023), this poses an environmental concern for the river basin water quality and potential bioaccumulation of metal(oid)s in the aquatic biota. Other mine wastes referred to as waste rocks (Figure 1e) are widespread in the area and consist of coarsely (mm in particle size) crushed low-grade ore or overburden discarded mining materials typically dumped in waste rock piles or used locally for construction purposes.

The Raibl ore deposit is located southward with respect to the “Periadriatic Lineament”, a regional W-E trending fault which divides the Adriatic from the European plate. Structurally, the Zn-Pb ore deposit is similarly controlled by Triassic to Early Jurassic extensional faults associated with the Pangea breakup in the extensional tectonic setting (Doglioni, 1988). In the area, the lithostratigraphic units belong to the Permian–Triassic (Figure 1a). The ore is hosted in thick and massive Ladinian dolomitic reefs: the Sciliar Formation or the so-called “Dolomia Metallifera”. It is a more than 1,000 m thick carbonate build-up, intercalated by local carbonatic-tuffaceous formations (Buchenstein Fm.). The Sciliar Formation is conformably overlain by the Carnian basinal bituminous limestones of the Calcare del Predil (Raibl Fm.) which act as a boundary for the ore deposit (Brigo et al., 1977; Jadoul et al., 2002).

Thallium (Tl) is a highly mobile and toxic metal in freshwater at very low concentrations ranging from a few μg/L to mg/L (Belzile & Chen, 2017; Campanella et al., 2019; Peter & Viraraghavan, 2005; Tatsi et al., 2015), and is found in two oxidation states, the most abundant monovalent Tl^+^ (Vink, 1993) and the most uncommon, toxic but less bioavailable trivalent Tl^3+^ (Ralph & Twiss, 2002). Thallium speciation, through Eh–pH diagram, in the water samples of the mine drainage from the Raibl mine (Barago et al., 2023) as well as from other mining sites (Casiot et al., 2011) suggest that the element in solution is expected to be in the dominant Tl^+^ form. Because of its similarity to potassium ions (K^+^), Tl^+^ is absorbed by plants and mammals through the skin and digestive and respiratory systems (Rodríguez-Mercado & Altamirano-Lozano, 2013). There is evidence of the effects of Tl on human health at doses well below the US-EPA maximum contaminant level in drinking water (2 µg/L). Despite this, there is still no specific Italian or European regulation of Tl in drinking water (Campanella et al., 2019 and references therein). At the Raibl mine, Tl is hosted in sulphide minerals such as sphalerite, especially of the yellow colloform variety, and in pyrite (Gartner, 2023; Melcher & Onuk, 2019; Pimminger et al., 1985; Schroll et al., 1994).

## Materials and methods

### Sampling strategy

**Fig. 2:**
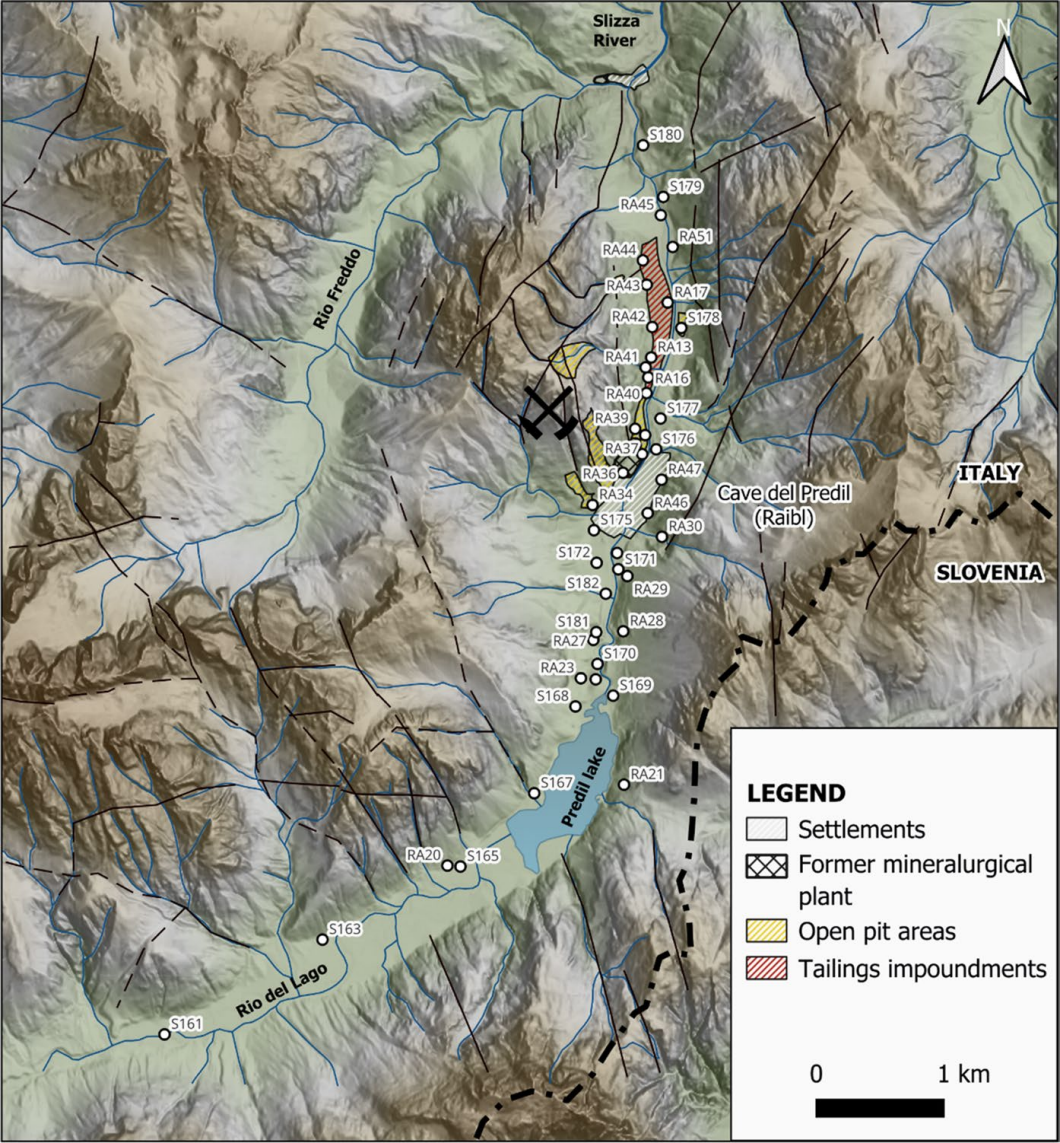
Map of the study area with the location of the sampling stations where soils and mine wastes (including rock wastes and tailings) were collected. Samples S161 and S163 were considered to be representative of the background values for the calculation of contamination degree (see the text for further explanation)

Sampling was performed within the Raibl mining area and along the Rio del Lago River valley from south to north with respect to the main entrance of the mine site and where the ore processing plant was located. A total of 44 samples including soil, waste rock and tailings (Fig. 2) were collected from the topsoil layer (30 cm) with a scoop. Removal of vegetation or plant litter from the topsoil was performed on site and rocky fragments larger than 2 cm were also removed using an appropriately sized sieve. Some soil samples near the mine entrance and the village were sampled in co-operation with ARPA FVG (Regional Environmental Protection Agency – Friuli Venezia Giulia, Italy). These samples are identified by the letter “S” in Fig. 2.

### Determination of organic matter (OM) content and pH

Samples were air-dried in the laboratory, sieved to 2 mm and ground using an agate mortar*.* Loss on ignition (LOI) allows one to rapidly evaluate the OM content in environmental samples (Heiri et al., 2001). Approximately ~ 1 g of sample was oven-dried at 105 °C overnight and then at 550 °C for 4 h in a furnace. Organic matter (OM) content was calculated according to the following equation:

$$OM \left( \% \right) = \left( { \frac{{m_{1} - m_{2} }}{{m_{1} }}} \right)*100$$

where m_1_ is the dry weight at 105 °C, and m_2_ is the dry weight at 550 °C.

Soil pH was determined on 1:2.5 soil to water suspensions. An aliquot of 5 g of each sample was placed in a conical flask and 12.5 mL of Milli-Q water were added and stirred on a magnetic plate for 2 h. The suspensions were subsequently centrifuged (ALC Centrifuge PK110) at 3,820 rpm for 10 min and pH (Table S4) was measured in the supernatant water by means of a portable probe (pH-meter CRISON PH25).

### Sample preparation and metal(oid) analytical determination

For total metal(oid) determination, approximately 250 mg of each sample were mineralised by acid-digestion in PTFE vessels through a total dissolution in a closed microwave system (Microwave PRO, Anton Paar) in accordance with EPA Method 3052 (US EPA, 1996) using a mixture of inverse *aqua regia* (HNO_3_ 65–69%: HCl 34–37% 3:1), HF 47–51% (VWR Normaton) and H_2_O_2_ (30 m/v, Merck, only in the case of soil samples). Two heating steps were performed and suprapure boric acid (H_3_BO_3_ 6%, Merck) was added in the second step to buffer the excess amount of HF. Blank samples and Certified Reference Materials (CRMs, PACS-3 and MESS-4 Marine Sediment Certified Reference Materials, NRCC, Canada) were also digested in each microwave batch and used to evaluate the accuracy of the procedure. After mineralisation, the solutions were diluted up to a volume of 25 mL by adding Milli-Q water and stored at 4 °C until analysis.

The determination of the labile and potentially bioavailable fraction of metal(oid)s was performed by means of a single-step PE using a 0.5 M HCl solution (Adami et al., 1999 and references therein) similar to other procedures reported in the literature (Hotton & Sutherland, 2016; Larner et al., 2006; Sutherland, 2002; Wang et al., 2017, 2020). For this purpose, 20 mL of the extracting solution were added to approximately 2 g of the finely ground sample. In addition, analytical blanks were also prepared, consisting solely of the extracting solution. The mixtures were stirred on a magnetic plate at room temperature for 18 ± 2 h, centrifuged (3,000 rpm for 15 min) and filtered (Millipore Millex HA, 0.45 µm pore size). The obtained solutions were diluted up to a final volume of 25 mL with Milli-Q water and stored at 4 °C until analysis.

Major elements such as Fe and metal(oid)s were determined by means of Inductively Coupled Plasma Mass Spectrometry (ICP-MS, NexION 350X Perkin Elmer equipped with an ESI SC autosampler). In order to avoid and minimise cell-formed polyatomic ion interference, the analysis was performed using Kinetic Energy Discrimination (KED) mode. The instrument was calibrated using standard solutions ranging between 0.5 and 500 µg/L prepared by diluting multistandard solutions for ICP analyses (Periodic Table MIX1 and MIX2, TraceCERT Sigma-Aldrich). Several aliquots of CRMs were analysed for total concentration to check for accuracy, and acceptable recoveries were obtained ranging between 81 and 119% (PACS-3) and 83–107% (MESS-4). Moreover, potential matrix effects were evaluated by means of laboratory-fortified samples prepared by spiking a standard solution different from that used for instrument calibration (Multi-element quality control standard for ICP, VWR Chemicals) into actual samples. Acceptable recoveries (ranging between 80 and 114%) were obtained thus indicating a negligible matrix effect. The precision of the analysis expressed as RSD was < 3%.

### Statistical analysis

Pearson’s correlation was used to assess the relationship between the various elements. Hierarchical cluster analysis was performed in R (R Core Team, 2024) by means of the Ward minimum variance method (Murtagh & Legendre, 2014) and Euclidean distance measures on the basis of the log-normalised values of 14 variables (i.e. the analysed trace elements).

The biplot associated with the principal component analysis (PCA) was calculated on log_10_ transformed data. The calculation was performed in Python via the Scikit-learn library (Pedregosa et al., 2011). Tests were performed to determine whether the data was fit for factory analysis: Bartlett’s sphericity test results were *p* < 0.001 and KMO equal from 0.61 to 0.70 (Kaiser–Meyer–Olkin test; acceptable values for KMO > 0.5). Data clustering later presented are also based on PCA results.

## Results and discussion

### Metal(oid) total concentration and spatial distribution

Total concentrations of metal(oid)s, including PTEs (i.e., As, Cd, Pb, Sb, Tl, Zn) and critical raw materials (i.e., As, Ge, Sb) are characterised by a very large variability, in the order of up to 10^4^ mg/kg (Table 1) due to the occurrence of different solid matrices (from soils to tailings). The samples were classified as reported in Table 1, taking into account similar characteristics on the basis of field information, OM content and PCA results which are described in paragraph 4.3.

**Table 1:** Range (min–max) of the total concentration (mg/kg), pH and OM (%) in different types of samples collected at the Raibl Pb–Zn mining site. Organic soil samples were considered on the basis of OM content > 50% determined through LOI method. The total element concentrations exceeding Italian regulatory limits are highlighted in bold. Column A: threshold limits for sites for public and residential use; Column B: threshold limits for sites for industrial and commercial use (Italian Decree 152/2006 according to EU Directive 2000/60/EC)

	Element	Soil	Organic soil	Waste rock	Tailings	Italian regulatory limits	
	n° samples	27	4	5	8	Col. A	Col. B
Total content (mg/kg)	As	1.27–**68.7**	7.06–**97.4**	**241**–**2,439**	**355**–**2,345**	**20**	**50**
	Cd	0.24–**16.0**	1.88–**18.4**	**8.14**–**196**	**4.25**–**40.2**	**2**	**15**
	Co	0.38–13.7	1.29–2.57	1.97–8.57	0.30–4.90	20	250
	Cr	3.22–45.9	14.2–20.8	12.1–59.6	2.07–54.9	150	800
	Cu	1.96–48.2	16.1–32.2	13.9–46.2	5.65–113	120	600
	Ge	< 0.50–2.86	< 0.50–3.52	11.3–92.7	10.1–92.4	\	\
	Fe	418–33,370	3,438–8,912	10,403–100,392	14,960–117,019	\	\
	Mn	29.0–1163	35.9–240	176–766	131–477	\	\
	Ni	1.76–14.7	7.26–10.0	9.17–20.5	3.44–26.0	120	500
	Pb	8.67–**4,387**	**145**–**2,625**	**3,903** – **50,062**	**1,704**–**19,542**	**100**	**1,000**
	Sb	0.09–6.04	1.25–3.96	3.00–**75.0**	2.04–**11.0**	10	30
	Tl	0.11–**14.5**	0.98–**19.0**	**30.0**–**907**	**65.1**–**754**	**1**	**10**
	V	4.45–69.1	16.5–31.4	18.2–73.0	7.89–65.0	90	250
	Zn	19.2–**4,456**	**182**–**4,586**	**9,462**–**114,965**	**9,566**–**60,824**	**150**	**1,500**
	pH	7.17–8.40	4.60–6.79	7.33–8.20	7.71–8.37	\	\
	OM (%)	3.75–39.5	58.4–84.9	5.31–50.0	6.93–18.7	\	\

**Fig. 3:**
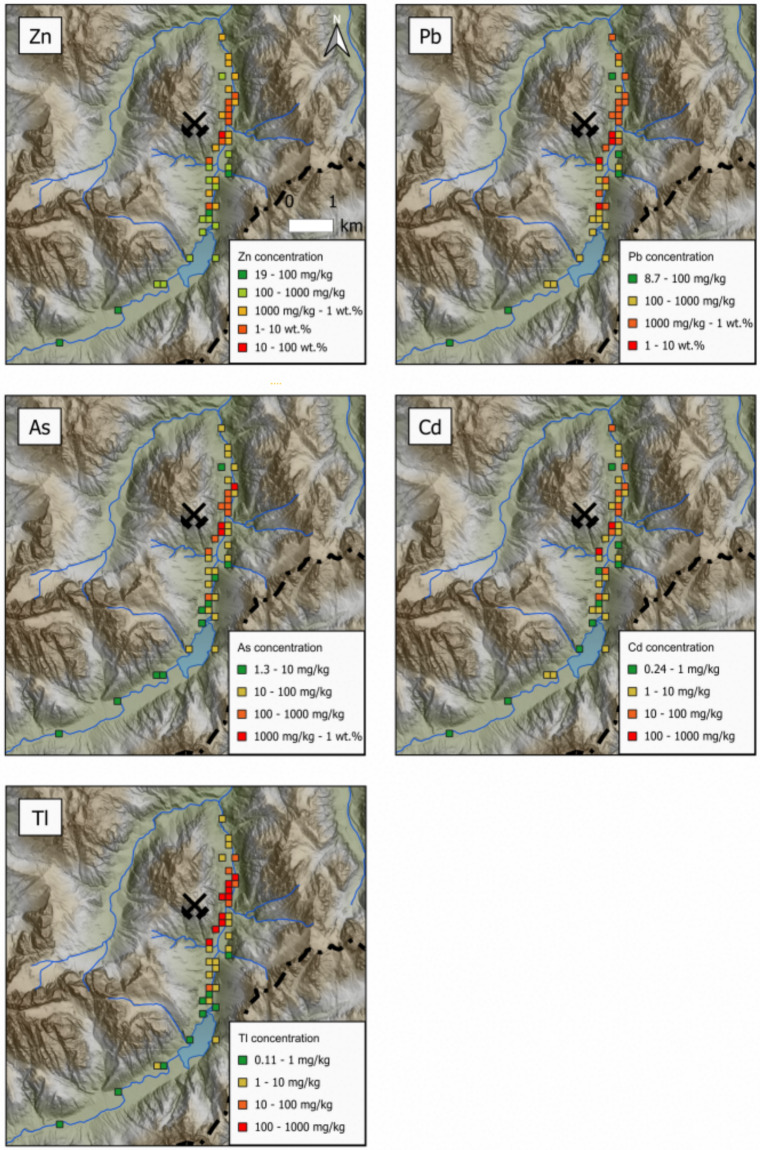
Total concentrations of Zn, Pb, As, Cd and Tl in soils and mine wastes (including waste rocks and tailings) at the decommissioned Zn-Pb Raibl mining site

Low to very elevated metal(oid) concentrations are dispersed around the mining village: up to around or over 100 mg/kg are observed for Tl, Sb, Cd, Ge; > 1,000 mg/kg for As; > 1% for Pb and > 10% for Zn and Fe. Generally, the lowest concentrations were observed upstream from the mining site, near Predil Lake which can be considered a pristine environment in the investigated area. To assess the contamination status in the area, the average concentration of two samples from this sector (S161 and S163, Fig. 3) were considered the background value for the calculation of the geoaccumulation index (I_geo_) according to the formula proposed by Müller (1969):

$$I_{geo} = \log_{2} \left( {\frac{{C_{n} }}{{1.5*B_{n} }}} \right)$$

where C_n_ is the measured concentration in the sample for the element “n”, B_n_ is the background value for the element “n” and the factor 1.5 is representative of potential variations of the background data due to lithological differences. The I_geo_ index consists of seven grades or classes from 0 (“uncontaminated”) to 6 (“very strongly contaminated”), with I_geo_ of 6 indicating an almost 100-fold enrichment above background values (Müller, 1969).

According to the I_geo_ index values (Table 2), 72% of the samples categorised as “soils” and “organic soils” (Table 1) were heavily contaminated (I_geo_ > 3) by Zn > Pb > Tl > As > Cd > Sb and among them 52% were extremely contaminated (I_geo_ > 5) by Zn > Pb > Tl > As. This is especially evident in the case of Zn, Pb and to a lesser extent Tl (Table 2), the latest showing I_geo_ values > 5 only in a few samples, mostly collected nearby or downstream from the tailings impoundments. On the contrary, mine waste samples were extremely contaminated, far exceeding the I_geo_ = 5 threshold especially in the case of Zn (maximum value of 10.6 and 11.5 in tailings and waste rocks, respectively), Pb (maximum value of 8.88 and 10.2 in tailings and waste rocks, respectively) and Tl (maximum value of 11.6 and 11.9 in tailings and waste rocks, respectively), followed by As, Cd and Sb (Table 2). Indeed, most of the enriched samples which frequently exceeded the Italian regulatory limits (Table 1) were found around mine waste piles made up of waste rocks and flotation tailings near the village of Cave del Predil among the mine entrance, the ore processing plant and the tailings impoundments, which are the sites where most of the mine wastes were allocated (Fig. 3).

**Table 2:** Average ± SD and maximum values of the geoaccumulation index (I_geo_) for As, Cd, Pb, Sb, Tl and Zn in different types of samples collected at the Raibl Pb–Zn mining site

	Element	Soil and organic soil	Waste rock	Tailings
	n	29	5	8
Geoaccumulation index (I_geo_)	As	2.51 ± 1.13 (max 5.10)	7.69 ± 1.27 (max 9.74)	8.41 ± 0.92 (max 9.69)
	Cd	1.72 ± 1.61 (max 4.63)	5.87 ± 1.90 (max 8.04)	4.55 ± 1.17 (max 5.76)
	Pb	3.43 ± 1.99 (max 6.72)	8.50 ± 1.76 (max 10.2)	6.83 ± 1.20 (max 8.88)
	Sb	2.49 ± 1.09 (max 4.58)	5.96 ± 1.95 (max 8.22)	4.46 ± 0.81 (max 5.45)
	Tl	3.29 ± 1.55 (max 6.26)	9.15 ± 1.76 (max 11.9)	10.3 ± 1.17 (max 11.6)
	Zn	3.91 ± 2.01 (max 6.89)	9.34 ± 1.52 (max 11.5)	9.10 ± 0.86 (max 10.6)

I_geo_ ≤ 0 uncontaminated; 0 < I_geo_ ≤ 1 uncontaminated to moderately contaminated; 1 < I_geo_ ≤ 2 moderately contaminated; 2 < I_geo_ ≤ 3 moderately to heavily contaminated; 3 < I_geo_ ≤ 4 heavily contaminated; 4 < I_geo_ ≤ 5 heavily to extremely contaminated; I_geo_ > 5 extremely contaminated

**Fig. 4:**
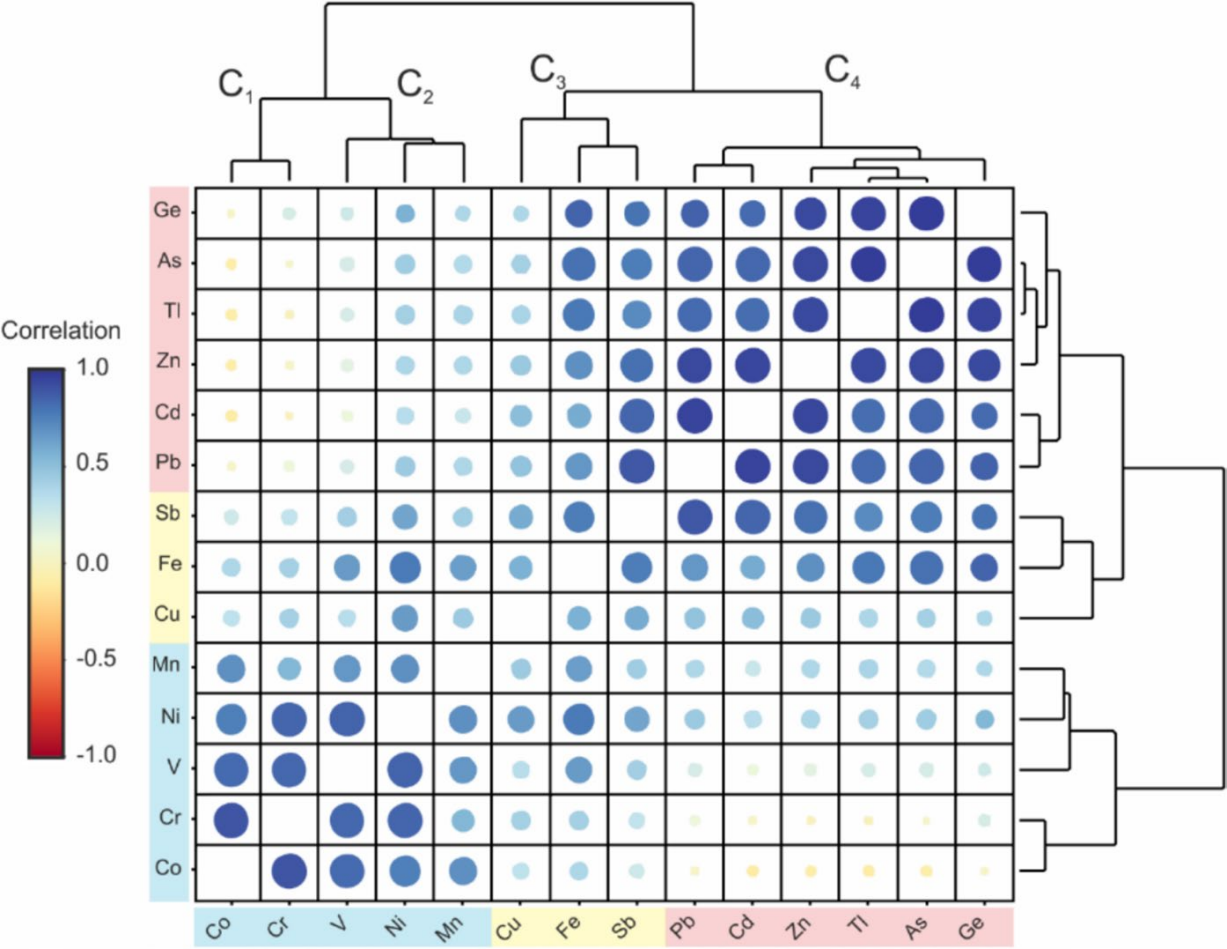
Pearson’s correlation clustermap of major and trace element total concentrations in all samples (processed using the heatmaply package; Galili et al., 2018). Point colour is a function of the correlation coefficient (r); point size is the log function of the *p*-value. Cluster C_1_ and C_2_ (blue) correspond to non-ore elements, C_3_ (yellow) to both ore and non-ore elements and C_4_ (red) to potentially toxic and critical elements

Three groups of variables (C1 + C2, C3 and C4) can be identified on the basis of the clustered Pearson’s correlation matrix obtained from the total concentrations (Fig. 4): (1) the Co-Cr-Mn-Ni-V group is associated with host rock and non-ore minerals of Triassic lithologies, basinal sediments and sporadic volcanic rocks; (2) the Cu-Fe-Sb group belongs both to host rock and metallic ore minerals such as, e.g., pyrite, HFOs and 3) the Zn-Pb-As-Cd-Ge-Tl group is related to ore minerals and includes both PTEs and critical elements. These metal(oid)s are associated with the primary (sphalerite, galena, pyrite) and secondary (hydrozincite, smithsonite, cerussite and HFOs) minerals. Due to their occurrence in the exploited ore body, samples with concentrations of these elements exceeding the Italian regulatory limits were found within all the categories listed in Table 1. For this reason, considerations regarding mobility are mainly limited to this group together with Fe as an important constituent of minerals still occurring in mine tailings (Table S1).

#### Mine wastes: waste rocks and tailings

In the study area, mine wastes are recognisable due to a lack of vegetation, the high presence of mineralised clasts in waste rocks and/or fine red–orange grain milled sediments (tailings). Waste samples are characterised by extremely elevated metal(oid) concentrations up to: Zn = 11.5%, Pb = 5.01%, As = 2,439 mg/kg, Tl = 907 mg/kg, Cd = 196 mg/kg and Ge = 92.4 mg/kg (Table 1). Concentrations are orders of magnitude higher than national regulatory limits for As, Cd, Pb, Tl and Zn (Table 1) and elevated concentrations are generally widespread in the entire area surrounding Cave del Predil. However, elevated concentrations were mainly observed downstream from the mine area, especially near the open pit mine areas, the former ore processing plant and the tailings impoundments.

#### Soils

Soil samples were divided into two classes on the basis of OM (%) calculated through the LOI method which is a proxy for the organic content: soils (OM < 50%) and organic soils (OM > 50%). Only 4 out of 31 soil samples showed an OM content > 50%. In general, concentrations of metal(oid)s in soils were orders of magnitude lower than those observed in mine wastes, although often still above the national regulatory limits (Table 1). The high variability of the spatial distribution of concentrations mainly depends on: 1) disseminated waste rock piles and waste rocks mixed with soils including as a result of their use for construction purposes (e.g. roads, service areas) and 2) aeolian dispersion of fine-grain tailings dust from the top of the impoundments during past spilling operations of the slurry and before they were capped (Stovern et al., 2015), as also confirmed by personal communication with retired miners.

### Labile fraction of metal(oid)s

The total concentration of metal(oid)s is not always representative of the real threat to potential receptors; instead, the determination of the labile fraction best defines mobility and potential bioavailability, allowing for a preliminary risk assessment concerning sources of contamination (Adami et al., 1999; Chester & Voutsinou, 1981; Duzgoren-Aydin et al., 2011; Wang et al., 2017). The HCl leaching approach performed in this study allowed for the evaluation of the amount of the labile fraction representing the mobile metal(oid)-bearing phases which can be transferred more easily from the solid matrix to the aqueous phase.

The results show a wide range of extractable metal(oid) contents, from very weak to extreme leaching based on different elements and matrices (Table 3). In detail, notably elevated extractable concentrations were observed in waste rocks and tailings, up to 6.95% for Zn and 1.14% for Pb. For the other metal(oid)s the highest leachable concentrations in waste rocks and tailings were: Tl = 255 mg/kg, Cd = 84.9 mg/kg, As = 26.7 mg/kg, and Sb = 0.76 mg/kg (Table 3). Conversely, minimum extractable concentrations were found in soils and organic soils which were less directly impacted by mining activity.

**Table 3:** Range (min–max) of the extractable content (mg/kg) and related fraction (% of the total content) of the main metal(oid)s (n.d. = not determined)

	Element	Soil	Organic soil	Waste rock	Tailings
	n	27	4	5	8
Extractable content (mg/kg)	As	< 0.01–1.07	0.52–26.7	0.38–3.77	< 0.01–0.15
	Cd	0.13–5.07	1.88–14.1	6.82–84.9	2.46–30.2
	Fe	8.98–2,373	293–1,576	144–3,088	2.74–31.6
	Pb	0.07–261	39.1–986	161–11,378	59.7–5,559
	Tl	< 0.01–1.10	0.09–4.76	1.44–21.2	11.4–255
	Zn	0.36–1,340	161–2,561	4,445–69,535	3,026–47,084
Mobile fraction (%)	As	n.d.–4.23	7.37–27.4	0.02–1.00	n.d.–0.01
	Cd	20.5–83.3	71.5–99.8	21.0–83.8	41.6–75.2
	Fe	0.05–7.11	7.33–21.8	0.68–7.87	< 0.10
	Pb	0.01–68.8	27.0–68.7	4.12–59.0	3.05–28.4
	Tl	n.d.–10.9	3.50–25.1	2.25–9.21	7.58–46.1
	Zn	0.10–35.2	45.7–88.6	22.3–75.6	28.3–77.4

**Fig. 5:**
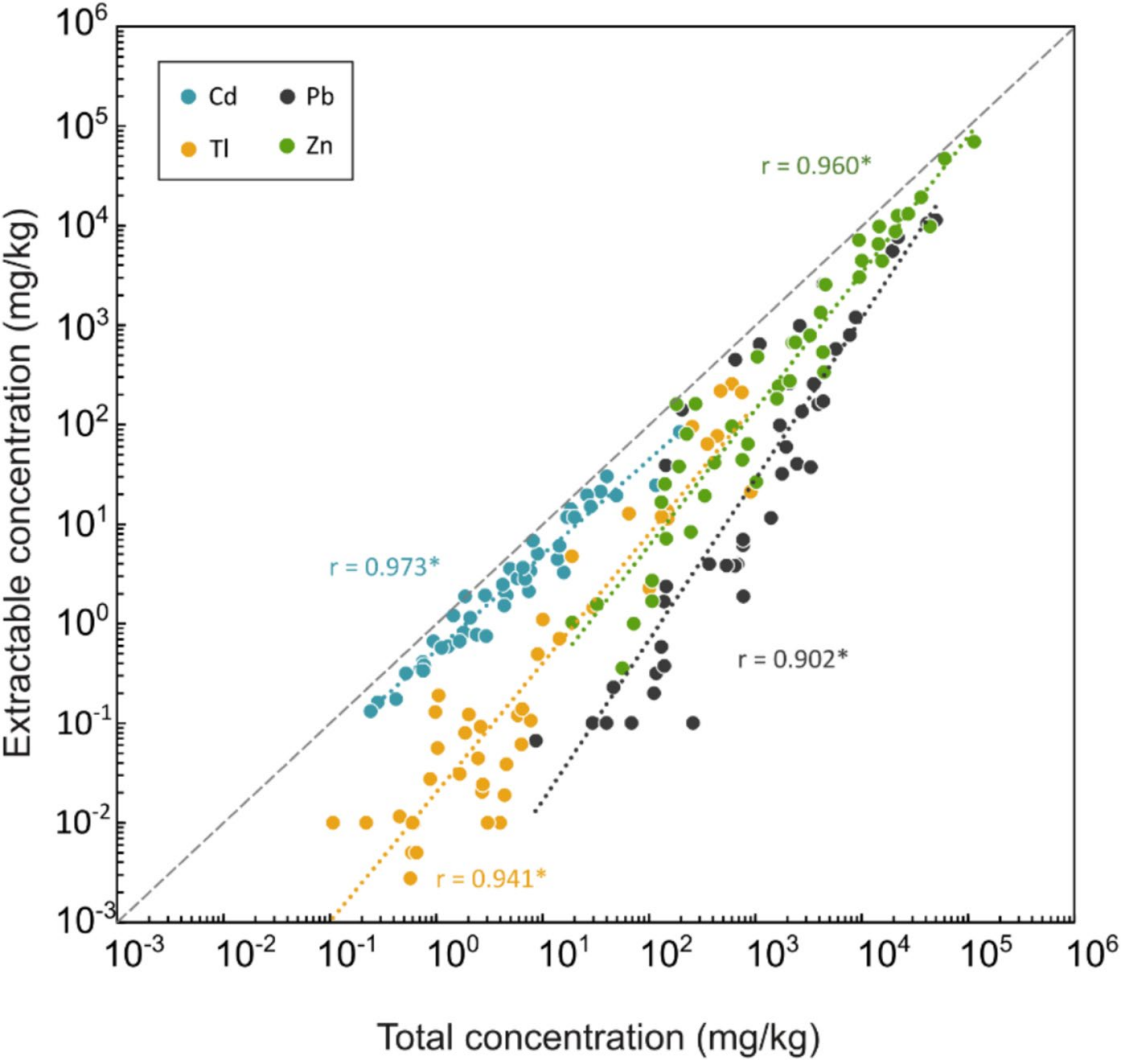
Relationship between diluted HCl extractable concentration and total content of Cd, Pb, Tl and Zn. * Pearson *p* < 0.001

Taking into consideration the more acidic laboratory physico-chemical conditions compared to those occurring in the natural environment, it was observed that Cd is generally the most mobile metal(oid) at the Raibl Zn-Pb district, showing the highest mobile fractions in all the analysed matrices, followed by Zn, Pb and Tl. Iron (Fe) and As were found to be the less mobile elements in this environment, in agreement with previous hydrogeochemical in situ observations (Barago et al., 2023). Extractable concentrations and fractions of Pb, Tl and Zn are indeed significantly correlated with total concentrations (Fig. 5) (*p* < 0.001, on log_10_ transformed data). A different behaviour was observed for Cd, since extractable concentrations increase with total concentrations, whereas its mobile fraction remains constantly elevated (between 20 and 100%, Table 3).

In general, mine wastes (waste rock and tailings) are characterised by the highest metal(oid) concentrations and also have the higher potential of metal(oid) release in solution. For instance, Tl extractable content is orders of magnitude higher in mining wastes than soils, since concentration values fall between 1.44 to 255 mg/kg (mobile fraction: from 2.25 to 46.1%) in mine wastes and between < 0.01 and 4.76 mg/kg (mobile fraction: from 0.43 to 10.9%) in soils (Table 3). The other metal(oid)s associated with the primary sulphide ore behave similarly: Zn extractable concentrations in mine wastes range from approximately 4,445 mg/kg to 69,535 mg/kg (mobile fraction: from 22.3 to 75.6%), whereas in soils they fall between 0.36 and 1,340 mg/kg (mobile fraction: from 0.10 to 35.2%). A similar behaviour was observed in the case of Pb, with extractable concentrations in mine wastes between 59.7 and 11,378 mg/kg (mobile fraction: from 3.05 to 59.0%) (Fig. 5).

**Fig. 6:**
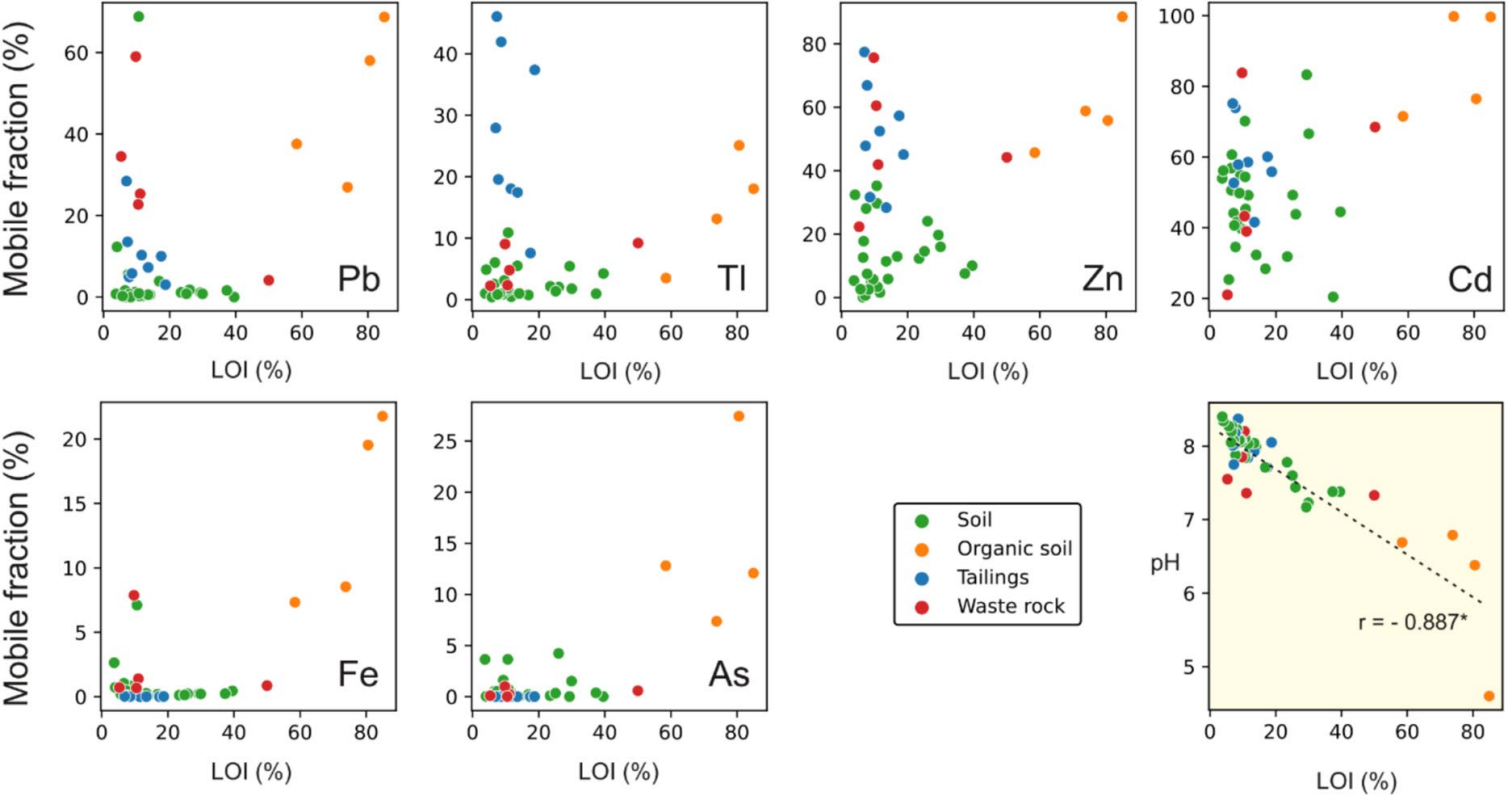
Relationships between the mobile fraction of Pb, Tl, Zn, Cd, Fe and As and OM as LOI (%), and between pH and OM as LOI (* Pearson, *p* < 0.001)

In contrast to Cd, Pb and Zn, Tl was found to be much more mobile in tailings than in waste rocks, both as extractable content and the labile fraction (Table 3 and Fig. 6). A few soil samples showed different characteristics than the other samples included in the dataset. Those samples, characterised by a high OM content (> 50%), were considered “organic soils”. Organic soils are also more acidic (pH from 4.60 to 6.79), as confirmed by the significant correlation between OM and pH (r = − 0.887, *p* < 0.001, Fig. 6) and are characterised by a higher mobile fraction of Pb, Tl, Zn, Cd together with Fe and As, which were found to be residual elements in the other sample matrices (soils, rock wastes and tailings) (Fig. 6). In these matrices, the lower labile fractions found for all metal(oid)s compared to organic soils may be related to the buffering effects of carbonates, which prevent the establishment of highly acidic conditions during the extraction with diluted HCl and thus limits the desorption from HFOs and the dissolution of sulphides. This matrix effect on the amount of metal(oid) extractable fractions can be confirmed by the pH values measured in the final extracts after treatment, which was still < 1 for organic soils and ~ 5 for soil, waste rock, and tailings samples.

### Classification of mine samples

**Fig. 7:**
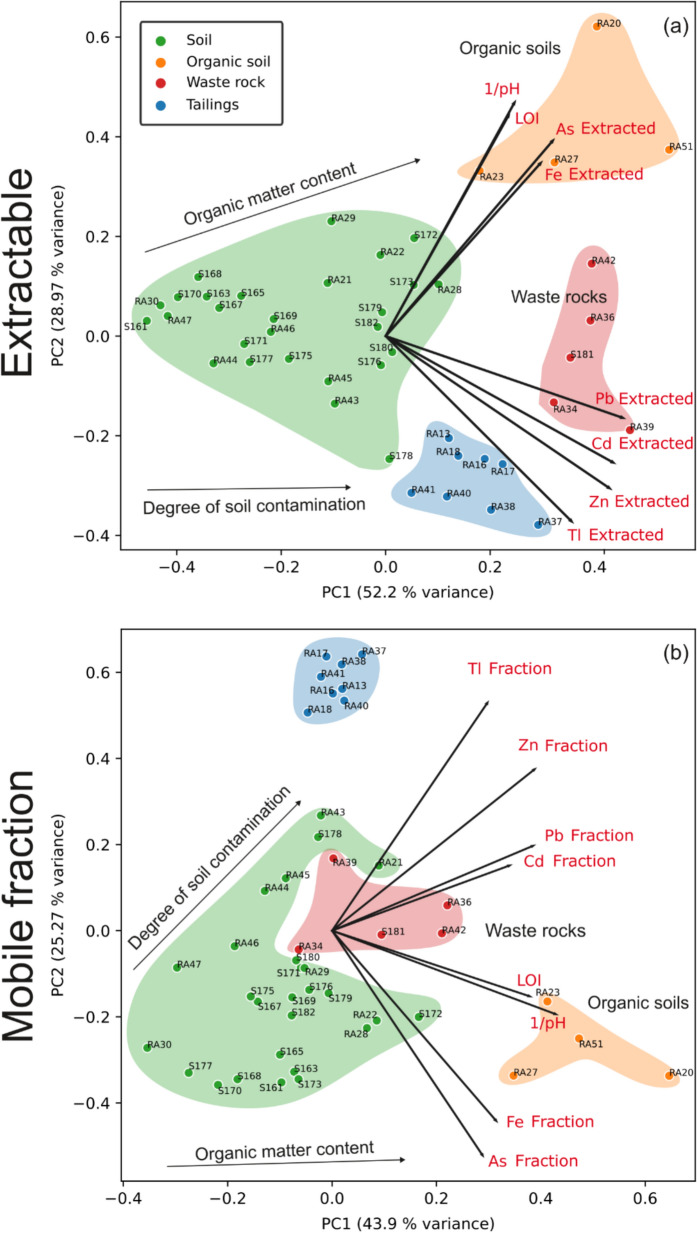
Biplots of extractable content in mg/kg (a) and mobile fractions in % (b) of soils, organic soils and mine wastes (rock wastes and tailings) from the Cave del Predil area

The classification of samples in a decommissioned mining area is necessary for the optimal management of mine wastes and is a challenging task as it can be difficult to determine whether samples belong to natural soils, tailings or waste rocks, or are a mixture of the three matrices. In this study, samples were also classified using multivariate analysis (Fig. 7) (Navarro-Murillo et al., 2024). Differences and similarities among samples which fall into four main groups (soil, organic soil, waste rocks and tailings) were shown in the PCA results (biplots, Fig. 7). The organic soil group is characterised by elevated OM and low pH, together with elevated Fe and As extractable contents and fractions. The waste rock group includes samples with the highest potential for leaching Pb, Cd and Zn, whereas the tailings group represents samples which mostly contribute to the release of Tl and Zn. The soil group includes samples that are, in fact, a mixture of natural soil with varying OM content and thus a degree of maturity, or more or less contaminated by waste rock and tailings.

### Impact of metal(oid)s on the environment and potential natural attenuation processes

Overall, the elevated concentrations found in environmental samples collected within the Raibl mining district indicate that natural soils as well as mining residues (including rock wastes and tailings) in this area were heavily impacted by As, Cd, Pb, Tl, Zn and Fe. This is also confirmed by the extremely elevated values obtained for the I_geo_ index, which testify to a strong degree of contamination for most of the investigated soils as a result of mixing with mine wastes. Moreover, results from the moderate extraction procedure reveal that most of the mineralised mine wastes may be potential sources of leachable Cd, Pb, Tl and Zn, which can be readily transferrable to surface waters and groundwater, as already observed in a previous study focused on the same area (Barago et al., 2023). When not properly managed, fine-grain size tailings may be a secondary source of metal(oid)s which are easily dispersed through runoff or wind deflation into the environment, threatening terrestrial and aquatic ecosystems. Due to the elevated concentrations in mine wastes widely dispersed within the study area (30–907 mg/kg), Tl may be easily uptaken by plants and bioaccumulated in wild plants and crops as well as transferred to the higher levels of the trophic web and, eventually, become a notable threat to human health (Xiao et al., 2004). Hyperaccumulation of Tl (and, to a lesser extent, Pb and Zn) in the shoots of some metallophytes adapted to live on metal-rich substrates has previously been reported near the Raibl mine, highlighting the strong impact of soil contamination on plant physiology in this area (Fellet et al., 2012).

Generally, the buffering effect of carbonate parent rock material in the study area prevented the acidification which can occur due to oxidation and hydrolysis reactions of metal sulphides derived from mining activity exposed to air and water (Walker et al., 2004). As a result, most of the samples showed pH values from neutral to alkaline, preventing acid mine drainage (AMD). Conversely, the OM remineralisation process and the presence of acid root exudates in organic soils have likely led to the observed decrease in pH values due to the progressive saturation of carbonate buffer capacity (Goulding, 2016; Hinsinger et al., 2003). In those soils which showed a large degree of maturity, in terms of OM content, higher mobile fractions of Zn, Pb, Cd, Tl together with Fe and As were found. It is known that at a lower pH, the mobility of metal(oid)s is generally higher due to the tendency to form more soluble ions such as Cd^2+^ , Zn^2+^ , Pb^2+^ and Tl ^+^ (Coup & Swedlund, 2015; Ford et al., 2007), and due to scarce adsorption on soil surfaces and competition with H ^+^ for binding sites (Kicińska et al., 2022; Rieuwerts et al., 2006). The occurrence of Fe in extracted solution suggests that more acidic conditions also promote the dissolution of metal(oid)-bearing Fe minerals. This may explain the higher amounts of metal(oid)s observed in the labile fraction of organic soils. Another aspect to be considered is the potential complexation of metal(oid)s by OM, which can affect metal(oid) mobility in the soil environment through the formation of soluble or insoluble complexes (Du Laing et al., 2009). This effect depends both on the quality of OM occurring in soils and the affinity of the different organic fractions for the different metal(oid)s and is in turn influenced by pH (Egli et al., 2010; Pérez-Esteban et al., 2014; Wong et al., 2007).

The increased mobility of metal(oid)s from organic soils may lead to an enhanced potential bioaccumulation in plants and mammals in the terrestrial environment (Buch et al., 2021; Pavoni et al., 2017) and, eventually, in fish in aquatic systems (Blasco et al., 1999; Mazej et al., 2010). Moreover, since soils near Cave del Predil are used for local crop cultivation and grazing, this enhanced metal(oid) mobility represents a potential concern for local populations. Indeed, instead of acting as a natural attenuation process, results suggested that soil ageing acts as a negative factor promoting the mobility of metal(oid)s with several potential negative effects such as higher bioaccumulation, leaching and transfer to other environmental compartments (i.e. water bodies, biota).

Following the extraction procedure with 0.5 M HCl, Tl appears to be less mobile than most of the other analysed metal(oid)s such as Cd, Co, Mn, Ni, Pb and Zn. A possible explanation could be that laboratory extraction with diluted HCl is representative of the most critical conditions in terms of metal(oid) mobility and may not entirely reflect the natural physico-chemical conditions found at the Zn-Pb mining site, characterised by slightly alkaline pH values due to the buffering effect of carbonates. For this reason, the mobility of some metal(oid)s as in the case of Pb, which was found to be more extractable than Tl at lower pH (Martínez & Motto, 2000), may indeed be overestimated and should be further investigated. Indeed, from in situ observations in neutral mine drainage (NMD) systems and laboratory tests, it was observed that Tl is more mobile and easily desorbed from HFOs than Pb (Barago et al., 2023; Coup & Swedlund, 2015). In this context, further experiments should be performed under natural pH conditions to evaluate the behaviour of Tl in slightly alkaline environments (NMD) and to properly assess its uptake by various plant species within the study area, which is not only dependent on total concentration (Jakovljević et al., 2024). Previous investigations in the area underline elevated concentrations of dissolved Tl in surface water and a higher dissolved Tl/Zn ratio was observed in the groundwater entrapped in the tailings impoundments (Barago et al., 2023). This anomaly was explained by a greater mobility of Tl compared to Zn, Pb, Cd (and other less mobile metal(oid)s) due to a particular hydrogeochemical behaviour that limits the natural attenuation processes of Tl (i.e. (co-)precipitation or sorption on HFOs). Tailings are characterised by comparable total concentrations of Tl (65.1–754 mg/kg) compared to waste rocks (30.0–907 mg/kg) and other sample matrices (Table 1). Conversely, the extractable concentrations in tailings (11.4–255 mg/kg) are one order of magnitude higher than those observed for waste rocks (1.44–21.2 mg/kg) as well as the percentage of mobile fractions (7.58–46.1 and 2.25–9.21% in tailings and waste rocks, respectively) (Table 3). Two possible reasons can be given to explain such a high Tl mobility. The main reason is that compared to other sample matrices, tailings are characterised by higher Tl total concentrations, which is positively correlated with its extractable concentrations (Fig. 5). Thallium (Tl) together with As are hosted in both sphalerite and pyrite (Gartner, 2023), and tailings are depleted in sphalerite and galena due to past ore processing, and relatively enriched in discarded pyrite and associated products of alterations of Fe sulphides, i.e. HFOs (Barago et al., 2023). In addition, the metal(oid)s predominantly associated with sphalerite and galena, such as Zn, Cd and Pb, respectively, are less abundant in tailings with respect to waste rocks, whereas the opposite holds true for As and Tl. For these reasons, it is reasonable to assume that an important contribution of labile Tl may be associated with the release of the element from pyrite and associated HFOs, as initially proposed by Petrini et al., (2016).

## Conclusions

As a result of past Zn-Pb mining, elevated concentrations of metal(oid)s, including PTEs, are widespread throughout the area of the former Cave del Predil mine due to the discharge and dispersion of mine waste materials. The spatial distribution of metal(oid)s and their leaching potential is influenced by both their occurrence and partitioning in parent rock material and the metallurgical processes to which the extracted ore was subjected. Among the investigated metal(oid)s, the group containing Zn-Pb-As-Cd-Ge-Tl is related to the ore and the associated mine waste, which includes both PTEs and critical and strategic elements such as Ge and As, which can be found in very high concentrations. In detail, these metal(oid)s can occur in very low to very elevated concentrations scattered throughout the tailings deposit area and the mining village in soils and mine wastes. Conversely, the lowest concentrations are observed upstream from the mining site, near Predil Lake, which may be considered a pristine and uncontaminated area.

The results obtained from partial extraction along with the OM content and pH helped in the classification of the samples, thus identifying soils, organic soils, waste rocks and tailings as well as soils mixed with the previous sample types. Among the investigated samples, mine wastes release the highest amounts of Zn, Pb, Tl and Cd and tailings are the main source of leachable Tl in the environment. Indeed, tailings are enriched in the more oxydisable pyrite and HFOs (i.e. providing Tl and As) and relatively depleted in sphalerite and galena (i.e. carriers of Zn and Pb) compared to waste rocks, due to industrial separation. In this context, the behaviour of Tl and other metal(oid)s is highly dependent on laboratory and field pH-Eh conditions which can be very different. Cadmium (Cd) was mobile at any concentration, although showing lower extractable contents than the other metal(oid)s. Arsenic and Fe were mainly mobilised in very rich organic soils (OM > 50%). Moreover, Cd, Zn, Pb and Tl were also found to be highly mobile in organic (and low acidic) soils. In this context, the occurrence of soluble organometallic complexes is not excluded. Interestingly, it seems that natural attenuation during ageing does not stabilise metal(oid)s and, conversely, increases their mobility. These aspects should be further investigated to better explain the influence of acidification and complexation by OM on the behaviour of metal(oid)s in soils of the investigated mining area as well as potential impacts on local ecosystems.

Such elevated concentrations and wide dispersion of metal(oid)s found in the area suggest that complete remediation may not be a sustainable choice. Future investigations should be focused on: 1) characterisation of the mine wastes accumulated in the tailings impoundments and evaluation of reprocessing approaches, not only to reduce contamination of water resources and health risks, but also to potentially recover critical and strategic elements such as Ge and As, as well as base metal(oid)s. Eventually, residual material should be stored as backfilling within the mine; 2) ecological and health assessments should be conducted on plants and livestock to evaluate a potential bioaccumulation related to human consumption as well as human screening to identify the main health risks for local communities and propose further mitigation actions.

## Supplementary Information

Below is the link to the electronic supplementary material.

Supplementary file1 (DOCX 375 KB)

Supplementary file2 (XLSX 42 KB)

## Data Availability

No datasets were generated or analysed during the current study.
