# Supplementary material for: Environmental impact and mobility of thallium and other metal(oid)s in soils and tailings near a decommissioned Zn-Pb mine (Raibl, NE Italian Alps)
Source: Environ Geochem Health. 2025 Feb 25;47(3):89. doi: 10.1007/s10653-025-02400-4 (PMC11861154; doi:10.1007/s10653-025-02400-4)
Supplement: Supplementary file 1 — Supplementary file1 (DOCX 375 KB) [file 10653_2025_2400_MOESM1_ESM.docx]

**Supplementary material**

**Table S1** – Mineralogical distribution of heavy minerals in a tailings sample from Raibl via SEM-EDS and EMPA analysis. The results do not include “light” minerals such as e.g. calcite and dolomite.

| **Mineralogy** | **N° of observations** | **Fraction (%)** |
| --- | --- | --- |
| Secondary Fe minerals (HFOs) | 250 | 70.0% |
| Secondary Zn minerals (smithsonite/hydrozincite) | 67 | 18.8% |
| Baryte | 21 | 5.9% |
| Fe sulfides (pyrite/marcasite) | 8 | 2.2% |
| Secondary Pb minerals (cerussite) | 7 | 2.0% |
| Silicates | 4 | 1.1% |
|  |  |  |
| Total | 357 | 100% |


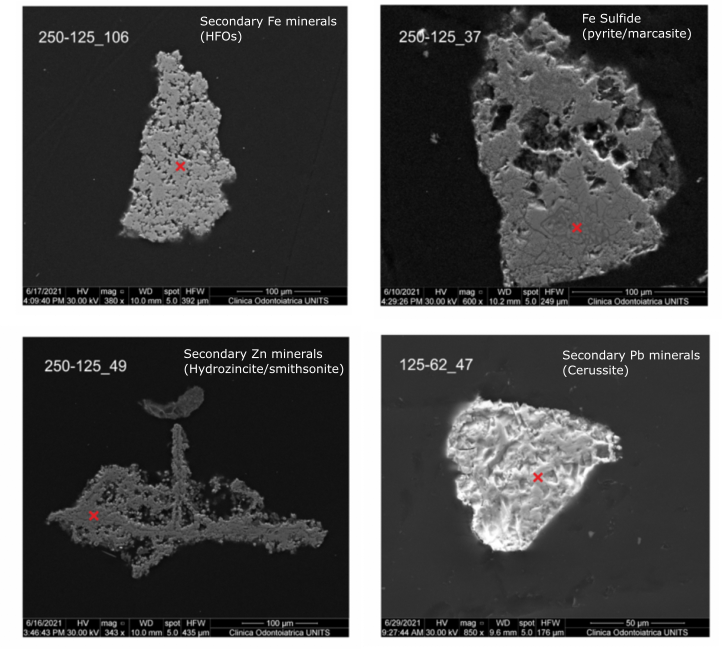


**Fig. S1** some examples of SEM analysis on tailings as per the results reported in Table S1 from Barago (2023).

**ICP-MS QA/QC**

ICP-MS was calibrated using standard solutions ranging between 0.5 and 500 µg/L prepared by diluting multistandard solutions for ICP analyses (Periodic Table MIX1 and MIX2, TraceCERT Sigma-Aldrich). Several aliquots of CRMs were analysed for total concentration to check for accuracy, and acceptable recoveries were obtained ranging between 81–119 % (PACS-3) and 83–107 % (MESS-4) were obtained. Moreover, potential matrix effects were evaluated by means of laboratory-fortified samples prepared by spiking a standard solution different from that employed for instrument calibration (Multi-element quality control standard for ICP, VWR Chemicals) into actual samples. Acceptable recoveries (ranging between 80 and 114 %) were obtained thus indicating a negligible matrix effect. The precision of the analysis expressed as RSD was < 3 %.
